
JOURNAL INFORMATION
==============================
NLM Title Abbreviation: ScientificWorldJournal
Journal ID: ScientificWorldJournal
NLM Journal ID: 101131163
Journal ID: TSWJ

The Scientific World Journal

ISSN: 2356-6140
EISSN: 1537-744X
Publisher: Wiley

ARTICLE INFORMATION
==============================
PMCID: PMC6084097
DOI: 10.1100/tsw.2001.448
Article ID: 375792
Article version: 1
Subjects: Extended Abstract

The CGRP-Antagonist BIBN4096BS Discriminates between the Action of α- and β-CGRP In Vitro

Wu Dongmei
Arnst Kirsten
Doods Henry
Boehringer Ingelheim Pharma KG, Cardiovascular Research, 88379 Biberach, Germany
Publication date: 2001
Electronic publication date: 2001 Oct 30
Volume: 1
First page: 49
Last page: 49
Copyright: Copyright © 2001 Dongmei Wu et al.
Copyright year: 2001
License: This is an open access article distributed under the Creative Commons Attribution License, which permits unrestricted use, distribution, and reproduction in any medium, provided the original work is properly cited.
License URL: https://creativecommons.org/licenses/by/3.0/

==============================
4th International Meeting on Calcitonin Gene-Related Peptide (CGRP)
*TheScientificWorld* (2001) 1(S1), 49
ISSN 1532-2246; DOI 10.1100/tsw.2001.448

### THE CGRP-ANTAGONIST BIBN4096BS DISCRIMINATES BETWEEN THE ACTION OF α- AND β-CGRP *IN VITRO*

Dongmei Wu, Kirsten Arndt, and Henry Doods
Boehringer Ingelheim Pharma KG, Cardiovascular Research, 88379 Biberach, Germany

Calcitonin gene-related peptide (CGRP) is a potent vasodilatory peptide implicated in migraine pathogenesis. Two protein isoforms, α-and β-CGRP, have been described and it has been reported that the two isoforms might activate two discrete CGRP receptor subtypes[1]. Recently, we showed that the nonpeptide CGRP antagonist BIBN4096BS can discriminate between putative CGRP-1 and CGRP-2 receptors in atrial and vas deferens tissue[2]. These data suggest that BIBN4096BS could be a useful tool in further characterizing CGRP-receptor heterogeneity. Therefore, in the present study we address the effects of BIBN4096BS on the action of α- and β-CGRP in four different tissues. Rat atria and vasa deferentia were employed as bioassays for CGRP-1 and -2 receptors, respectively. Results show that in rat atrium BIBN4096BS did not discriminate between effects of human α- and β-CGRP ($pK_B$ = 8.52 and 8.13). Similar results were obtained for hα- and hβ-CGRP in the vas deferens ($pK_B$ = 7.11). Interestingly, in pig cerebral and coronary arteries BIBN4096BS did discriminate between the action of hα and hβ-CGRP ($pK_B$ = 8.08/8.0 and 6.75/6.6) indicating a stronger inhibitory effect of BIBN4096BS on α-CGRP dilatory action compared to β-CGRP in the arteries. In conclusion, our results demonstrate BIBN4096BS as a highly potent and selective tool revealing CGRP-receptor heterogeneity.

### REFERENCES

1. Jansen, I. (1992) Neuropeptides 21, 73–79.
2. Wu, D. et al. (2000) Eur. J. Pharmacol. 400, 313–319.
